# An unusual case of dengue shock syndrome complicated by ilio-femoral deep vein thrombosis; a case report

**DOI:** 10.1186/s12879-020-05062-y

**Published:** 2020-05-12

**Authors:** K. M. I. U. Ranasinghe, D. Dissanayaka, K. Thirumavalavan, M. Seneviratne

**Affiliations:** 1 Registrar in Medicine, National Hospital of Sri Lanka, Colombo, Sri Lanka; 2 Physician, National Hospital of Sri Lanka, Colombo, Sri Lanka

**Keywords:** Dengue, Dengue shock syndrome, Deep vein thrombosis, Thrombocytopenia

## Abstract

**Background:**

Dengue fever is a hemorrhagic fever caused by flaviviruses. Hemorrhagic manifestations are well known to be associated with dengue fever, though the thrombotic events are only seldom reported. Underlying pathophysiology of thrombotic events is multifactorial and the management is challenging due to associated thrombocytopenia and bleeding tendency. We report a case of dengue shock syndrome with severe thrombocytopenia complicated by ilio-femoral deep vein thrombosis.

**Case presentation:**

A 16 year old boy presented with dengue fever. He had dengue shock syndrome after entering the critical phase on the fifth day of the illness. With the recovery from the critical phase he developed deep vein thrombosis involving right external iliac, common femoral and superficial femoral veins. There were no provocative factors other than dengue fever itself. His platelet count was 12,000/μl at the time of diagnosis with deep vein thrombosis. Anticoagulation was started with intravenous unfractionated heparin 500 IU/hour while closely being observed for bleeding complications. 1000 IU/hour dose was commenced with the recovery of the platelet count above 50,000/μl. Thrombophilia screening was negative and he was discharged on warfarin. Venous duplex done after 6 weeks showed normal lower limb venous flow and warfarin was omitted after three months.

**Conclusions:**

With dengue fever, complications like deep vein thrombosis can be easily missed given its rarity and that the major concern is on hemorrhagic complications. Management is challenging due to associated thrombocytopenia and hemorrhagic complications.

## Background

Dengue fever is a hemorrhagic fever caused by flaviviruses. Five antigenically related but distinct serovars have been identified, even though the strain 5 is not known to be associated with human infections so far. Bites of infected female mosquitoes *Aedes aegypti* and *Aedes albopictus* result in virus transmission. Incubation period is between 4 and 10 days [1]. It is endemic in many countries, highest in parts of Asia and South America.

In Sri Lanka, a total of 55,894 dengue cases and 74 deaths due to dengue fever have been reported within the year of 2019 as at October 18th. This is a significant increase of the disease burden in contrast to 2018 where only 58 dengue deaths were reported during the whole year [2]. Not confining to Sri Lanka, incidence of the disease has dramatically risen worldwide during the past few years making dengue fever, a major public health concern. WHO has declared that about half of the world population is now at risk of the disease [3].

Dengue causes a wide spectrum of diseases ranging from asymptomatic infection to severe flu-like symptoms. The World Health Organization classifies dengue into two major categories: dengue (with / without warning signs) and severe dengue. Severe dengue is characterized by plasma leaking, fluid accumulation, respiratory distress, severe bleeding, organ impairment and metabolic abnormalities [3, 4].

Hemorrhagic manifestations are well known to be associated with dengue. Thrombotic events complicating acute dengue illness are seldom reported and only case reports are found in literature [5–7]. There is a descriptive study done in Brazil where five patients were reported having deep vein thrombosis. All of them had dengue fever without evidence of plasma leakage. Three other reported cases, including two from Sri Lanka had deep vein thrombotic events following recovery from the initial infection, but none of them had severe thrombocytopenia (< 20,000/μl) during the time of occurrence of the thrombotic event [5, 6, 8, 9].

We report a case of a patient with dengue shock syndrome, who developed ilio-femoral deep vein thrombosis during the critical phase, despite severe thrombocytopenia of 12,000/ μl.

## Case presentation

A 16 year old Sri Lankan boy presented to the National Hospital of Sri Lanka, with a 4 day history of high grade fever, arthralgia, myalgia and headache. He was obese with a Body Mass Index of 28 kg/m^2^. NS_1_ antigen positivity confirmed the diagnosis of dengue fever. On the fifth day of the illness he went into the critical phase as evidenced by fluid in the hepato-renal pouch in bedside ultrasound scan. His platelet count was 52,000/μl at the time of entering the critical phase. Other hemodynamic parameters were stable and the urine output was within the desired range. By the 18th hour of the critical phase he went into compensated shock. Initial crystalloid bolus was given according to the national guideline for the management of dengue.

His hemodynamic parameters improved after 2 hours with a blood pressure of 100/75 mmHg and the urine output more than 0.5 ml/kg/hr. Packed Cell Volume (PCV) dropped to 46% from 51%. His baseline PCV on admission was 40%. He again went into compensated shock the next hour requiring another two crystalloid boluses. After maintaining hemodynamic parameters stable for 2 hours, he went into uncompensated shock and PCV was 46%. As the PCV rise could not explain the shock by fluid leakage alone, immediate red cell transfusion was arranged. He was given another two boluses of dextran consecutively followed by another 5 ml/kg red cell transfusion as hemodynamics did not improve. He responded gradually to the above management. During this period, other causes for poor response to fluid resuscitation, including ABCS, were excluded. Bedside ultrasound scan did not show any intra-abdominal bleeding. He did not have any other overt bleeding manifestation.

His lowest platelet count was 7000/μl, and that was on the first day of the critical phase. By the end of the second day of the critical phase it rose to 12,000/μl. On the third day after entering the critical phase, he complained of right sided leg swelling and pain. Examination revealed grossly swollen erythematous right lower limb with increased warmth and tenderness. Early convalescence rash was apparent. Clinical suspicion of deep vein thrombosis was confirmed by lower limb duplex scan, which showed deep vein thrombosis involving right external iliac, common femoral and superficial femoral veins.

As the latest platelet count by the time of diagnosis of deep vein thrombosis was 12,000/μl, we were in a therapeutic dilemma. Thromboembolism deterrent (TED) stockings were applied to the unaffected limb and until anticoagulation right lower limb was kept immobile. Hematology opinion was taken, after which anticoagulation with unfractionated heparin (UFH) was decided. Coagulation studies, PT/INR, APTT were within the normal range. While awaiting repeat full blood count, intravenous UFH 500 IU/hour was started. We observed him closely for bleeding. Platelet count had risen up to 58,000/μl in the next full blood count, after which UFH dose was increased to 1000 IU/hour. APTT was 48 s and 54 s at the 6th hour and the 12th hour of UFH respectively. Warfarin was commenced the next day and therapeutic range (INR 2–3) was achieved by the sixth day of the diagnosis of DVT.

As thrombotic events are rare in severe dengue illness, we looked for other thrombophilia conditions. He did not have any significant past history to suggest thrombotic events. Thrombophilia screening which included anticardiolipin antibodies, anti beta2 glycoprotein, protein C and S levels and prothrombin gene mutation was negative. Above investigations were arranged before starting UFH, as anticoagulation would affect the results otherwise. The patient was discharged after 12 days of hospitalization. He did not have any other complication of dengue fever apart from transient liver transaminitis which resolved before discharging. He was reviewed after 6 weeks from the onset of DVT and venous duplex showed recanalization of the right sided lower limb deep veins with a normal venous flow. Warfarin was omitted at the end of 3 months.

## Discussion and conclusions

Although hemorrhagic manifestations are well known to occur with dengue fever, only a few cases complicated with thrombotic events are reported. Up to now the largest number of reported cases is from Brazil. Ninety-two serologically confirmed patients were studied from January 2011 to March 2011, where five patients were given the diagnosis as having large vessel thrombosis representing 5.4% of all dengue patients. Thrombotic events reported were, two cases of ilio-femoral deep vein thrombosis, two cases of pulmonary embolism and a case of mesenteric vein thrombosis. All these events occurred within the first 5 days of admission, and the lowest platelet count reported was 37,000/microl. None of the affected patients were classified as having dengue shock syndrome or dengue hemorrhagic fever [5]. Three other reported cases of deep vein thrombotic events were found in the literature. All those patients developed deep vein thrombotic events after the recovery from the acute illness and the recovery of the platelet count [8, 9]. Another two cases of cerebral venous thrombosis were reported where dehydration associated with the illness was thought to be the causative factor [10, 11].

An arterial thrombotic event manifested as an ischemic stroke was reported in India in a patient with dengue fever. He did not have underlying conventional risk factors for an ischemic stroke. In addition to transient hypercoagulability of dengue fever, meningovasculitis was considered responsible for this occurrence [12].

Thrombus formation and propagation are caused by abnormalities of the blood flow, vascular endothelium and blood clotting components, collectively known as Virchow’s triad. In dengue hemorrhagic fever and shock syndrome due to plasma leakage, there is a significant hemoconcentration manifested by high PCV. During the initial period of shock our patient had PCV of 51%, whereas his baseline was 40% depicting significant hemoconcentration. However this amount of PCV rise by about 20% is not uncommon with severe dengue, and it does not cause thrombosis usually. Notably hypovolemic shock in our patient was not entirely due to plasma leakage, as the PCV did not rise as would be seen in case of shock only due to plasma leakage. He required red cell transfusions as well for the hemodynamic stability. The exact point of bleeding could not be identified, however concealed hemorrhages are well known to occur with dengue fever which could have been the case with our patient as well.

Factors associated with thrombosis in dengue fever are not well described in the literature. Myriad of factors including enhanced complement activation, cytokine storm due to antibody-dependent enhancement of infection and enhanced immune complex formation may increase thrombotic events in these patients. In the early course of severe dengue fever, loss of endothelial non-thrombogenic protective factors has been identified [13, 14]. Infected endothelial cells are known to express thrombomodulin facilitating coagulation [15]. Hemoconcentration during the critical phase, immobility during the hospital stay and usage of colloids in the management of shock will also enhance thrombogenecity in patients with dengue hemorrhagic fever.

A reduction in the level of activated protein C is seen in association with dengue viral infection probably due to down regulation of thrombomodulin-thrombin-protein C complex formation. In addition, low plasma concentration of protein S, tissue factor inhibitor and antithrombin III has been reported with severe dengue fever, though it is not associated with clinical thrombosis [14, 16, 17]. The levels of these factors are inversely proportional to the severity of shock and are likely caused by capillary leakage [14]. In addition, plasma concentrations of Plasminogen activator inhibitor type-I (PAI-1) are seen elevated with dengue infection [13].

An imbalance in the vWF-ADAMTS-13 system has been observed recently in association with dengue infection complicating the understanding of the pathogenesis of coagulopathy. In patients with severe illness low levels of ADAMTS-13 and high levels of elongated active vWF were discovered. Thus unregulated vWF due to low levels of ADAMTS13, leads to the platelet glycoprotein receptor Ib exposure to another platelet, facilitating platelet aggregation and the generation of platelet rich micro thrombi [18, 19].

As described above a constellation of factors may contribute to the development of thrombosis in a patient with dengue fever, rarely leading to major thrombotic events. This case describes a rare complication of dengue fever, deep vein thrombosis. Pulmonary embolism may cause or worsen hemodynamic instability complicating the condition further. Thus physicians’ awareness about this rare complication is of paramount importance in managing unusually complicated cases of dengue infection with fluid unresponsiveness.

## Data Availability

All necessary data and material are provided.

## Abbreviations

ABCS: Acidosis, bleeding, calcium, sugar
ADAMTS-13: A disintegrin and metalloproteinase with thrombospondin type 1 motif, member 13
APTT: Activated partial thromboplastin time
BMI: Body mass index
DVT: Deep vein thrombosis
PCV: Packed cell volume
PT/INR: Prothrombin time, international normalized ratio
TED: Thromboembolism-deterrent
UFH: Unfractionated heparin
vVF: Von Willebrand factor
